# Malakoplakia of the gastrointestinal tract: clinicopathologic analysis of 23 cases

**DOI:** 10.1186/s13000-020-01013-y

**Published:** 2020-07-24

**Authors:** Michael Lee, Huaibin Mabel Ko, Anthony Rubino, Hwajeong Lee, Ryan Gill, Stephen M. Lagana

**Affiliations:** 1 Department of Pathology and Cell Biology, Columbia University Medical Center, 630 West 168th Street, VC14-240A, New York, NY 10032 USA; 2 Department of Pathology and Laboratory Medicine, Mt. Sinai Medical Center, New York, NY USA; 3 Department of Pathology, Albany Medical Center, Albany, NY USA; 4 Department of Pathology and Laboratory Medicine, University of California-San Francisco, San Francisco, CA USA

**Keywords:** Pathology, Malakoplakia, Gastrointestinal tract

## Abstract

**Background:**

Malakoplakia is an uncommon, tumor-like inflammatory disease characterized by impaired histiocytes that are unable to completely digest phagocytized bacteria. The genitourinary tract is the most common site of involvement, however, cases have also been described in the gastrointestinal tract, suggesting that it is the second most common site of involvement. This study investigates the clinical and histologic features of malakoplakia in the gastrointestinal tract.

**Case presentation:**

For 23 gastrointestinal specimens (biopsies and resections) from patients with a pathologic diagnosis of malakoplakia, we recorded the gender, age, location, primary diagnosis, endoscopic or surgical indication, endoscopic/gross impression and immune status (immunocompromised vs. immunocompetent).

**Conclusion:**

Malakoplakia occurred throughout the length of the gastrointestinal tract with most of the cases located in the sigmoid colon and rectum (*n* = 10); other sites included the transverse and descending colon (*n* = 4), stomach/gastroesophageal junction (*n* = 4), appendix (*n* = 2), cecum (*n* = 1), small bowel (*n* = 1), and the peri-anal area (*n* = 1). Endoscopically, these lesions most commonly appeared as polyps (*n* = 10) or masses (*n* = 5), other clinical endoscopic impressions varied from a thickened area/fibrosis to mucosal erythema. Most patients were immunocompromised due to a disease state (e.g. organ transplantation, cancer diagnosis, autoimmune condition) and/or medication effect. Eight patients with malakoplakia were on immunosuppressive medications (8/23, 35%). Common immunosuppressed disease states included cancer (*n* = 9), autoimmune disease (*n* = 5), status post organ transplantation (*n* = 4), diabetes (*n* = 5), infection/sepsis (*n* = 3), and HIV/AIDS (*n* = 1). Some patients had multiple co-morbidities (i.e. diabetes and organ transplant). Twenty-one patients with malakoplakia were in an immunosuppressive state (21/23, 91%).

## Introduction

Malakoplakia is a rare inflammatory condition characterized by impaired macrophages with an inability to completely digest and kill bacteria. Macrophages phagocytize bacteria, however, partially digested bacterial components accumulate within the phagolysosome [1]. Possible theories for this dysfunction include an abnormal immune response, defective lysosome function with impaired bactericidal activity due to decreased cyclic guanosine monophosphate [2, 3] or a bacterial organism-specific process [1]. The most commonly implicated organism is *Escherichia coli* but *Klebsiella pneumoniae, Mycobacterium tuberculosis, Proteus, Rhodococcus equi, Staphylococcus aureus* and *Pseudomonas aeruginosa* have also been isolated from malakoplakia lesions [1, 4, 5]. It is likely a confluence of these factors that contribute to the pathobiology.

**Fig. 1:**
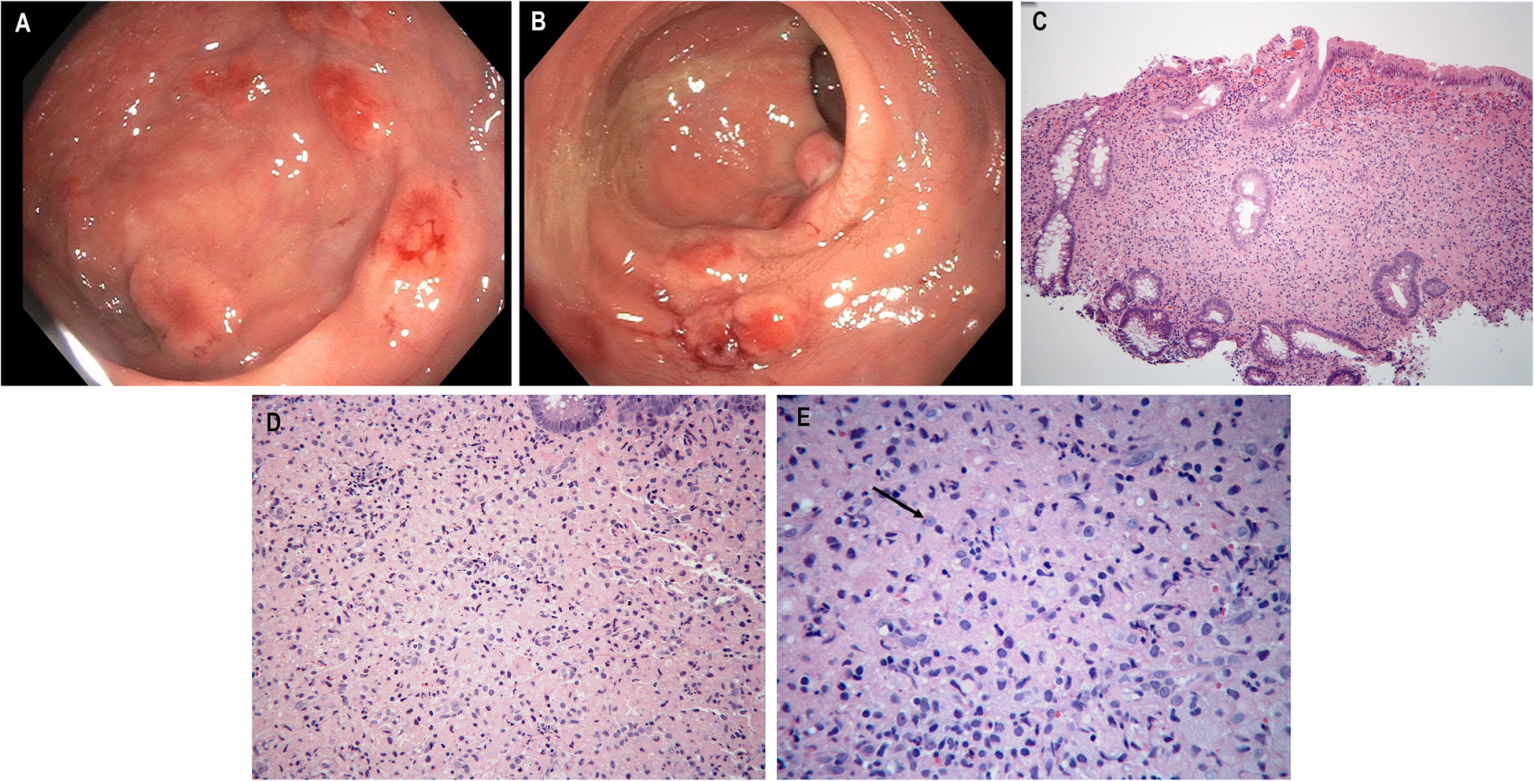
Multiple circumscribed nodules are seen in the rectum on colonoscopy (**a**). There is also bleeding and erythema of the mucosa which led to hematochezia (**b**). There is marked expansion of the lamina propria by a predominantly histiocytic infiltrate. Colonic crypts are distorted and pushed aside. H&E 200x magnification (**c**). Histiocytes are filled with eosinophilic debris and have smooth nuclear contours with small, prominent nucleoli. Scattered heterogenous inflammation composed of neutrophils, lymphocytes and eosinophils is seen in the background. H&E 400x magnification (**d**). Higher power demonstrates Michaelis-Gutman bodies (arrow). These pale, targetoid and lamellated inclusions are seen in the cytoplasm of the histiocytes. H&E 600x magnification (**e**)

While the name suggests malakoplakia would grossly appear as soft plaques (*malakos* meaning soft and *placos* meaning plaque), the clinical appearance in the gastrointestinal tract varies, ranging from polyps (Fig. 1 a, b), flat lesions, ulceration, mucosal erosions or plaques. Mass forming tumors mimicking malignancy have also been described [6–8]. Malakoplakia was initially described and most commonly found in the genitourinary tract but since its initial discovery in the early twentieth century, it has been reported in nearly every organ system, including the gastrointestinal [9–15], liver [16], central nervous system [17], renal [18], and pulmonary tract [19]. The gastrointestinal tract is the second most common organ system for malakoplakia involvement and it has been seen in association with colonic adenomas and adenocarcinomas [9, 20, 21].

Patients with malakoplakia are often immunocompromised, secondary to solid organ transplant, malignancy, acquired immunodeficiency syndrome, autoimmune disease or any clinical condition requiring immunosuppression and steroids [1, 22]. While malakoplakia of the gastrointestinal tract has been documented in the literature as case reports, this study is the first multi-institutional case series for this location. Since the gastrointestinal tract is the second most common organ system of involvement, knowledge of the full spectrum of clinical and histopathologic features will inform pathologists about this entity, especially in immunocompromised patients.

## Materials and methods

This study was a collaborative effort between departments of surgical pathology at Columbia University Medical Center, Mt. Sinai Medical Center, Albany Medical Center and the University of California–San Francisco. Cases were identified from the electronic medical records of these hospitals with institutional research board approval. The pathology archives were searched for biopsies and resections with a diagnosis of malakoplakia in the gastrointestinal tract from 2002 to 2019. A total of 23 cases were identified with corresponding hematoxylin and eosin stained slides, special slides and associated clinical information. For each patient, we recorded the age, gender, location, endoscopic impression, symptoms, medications, comorbidities, immunosuppressive state and other pertinent details.

## Results

Twenty-three total cases of malakoplakia involving the gastrointestinal tract were identified. The 23 patients included 11 males and 12 females, with a mean age of 57 years (range: 3.5 months to 92 years). Indications for endoscopy were available for 21 cases. The most common reason for upper endoscopy or colonoscopy was diagnostic screening (*n* = 7); other indications included diarrhea (*n* = 3), rule out rejection (*n* = 1), gastrointestinal bleeding (n = 1), abdominal pain (*n* = 1), and follow up of Barrett’s esophagus (*n* = 1). Seven cases were an incidental finding adjacent to a malignant tumor on surgical resection. Our case series showed malakoplakia occurring throughout the length of the gastrointestinal tract with the majority of cases in the sigmoid colon and rectum (*n* = 10); other sites included the transverse and descending colon (*n* = 4), stomach/gastroesophageal junction (n = 4), appendix (*n* = 2), cecum (*n* = 1), small bowel (*n* = 1), and the peri-anal area (*n* = 1). Endoscopically, these lesions most commonly appeared as polyps (*n* = 10, Fig. 1a, b) or masses (*n* = 5), other clinical endoscopic impressions varied from a thickened, fibrotic induration to mucosal erythema (Table 1).

**Table 1:** Results for malakoplakia of the gastrointestinal tract based on location, diagnosis and endoscopic impression

Location	Medical history/diagnosis	Endoscopic impression
Sigmoid and rectum (10)	Cancer (9)	Polyp (10)
Transverse/descending (4)	Autoimmune disease (5)	Mass (5)
Stomach/GE junction (4)	Diabetes mellitus (5)	Fibrotic thickening (3)
Appendix (2)	Organ transplant (4)	Erythema (3)
Small bowel (1)	Infection/sepsis (3)	Edematous (1)
Cecum (1)	HIV/AIDS (1)	Other (1)
Peri-anal (1)		

*GE* Gastroesophageal

**Fig. 2:**
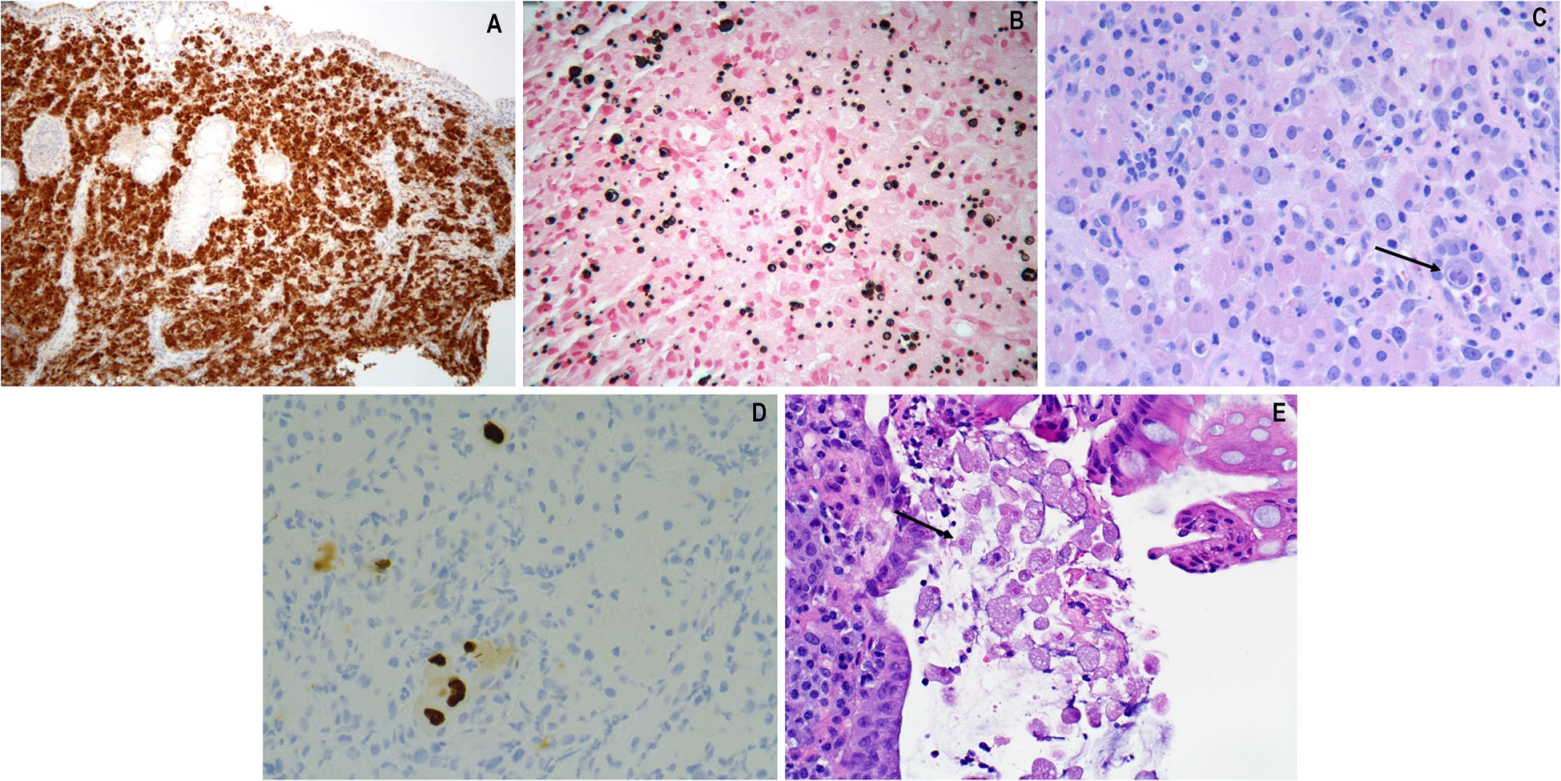
CD68 immunostain highlights the histiocytes in malakoplakia. CD68 immunostain 200x magnification (**a**). Von Kossa special stain highlights the calcified and lamellated Michaelis-Gutman bodies. Von Kossa special stain 400x magnification (**b**). Since immunocompromised patients are at risk for malakoplakia, concomitant cytomegalovirus infection (arrow) can be seen. H&E 400x magnification (**c**). CMV immunostain highlights intra-nuclear viral inclusions. CMV immunostain 400x magnification (**d**). *Entamoeba histolytica* is a protozoal parasite (arrow) with foamy cytoplasm, phagocytized red blood cell inclusions, and eccentrically located nuclei. These microorganisms were seen in a patient with malakoplakia. H&E 400x magnification (**e**)

The gross appearance is variable but nodularity or mass-like growth is common. Microscopically, malakoplakia demonstrates histiocytic aggregates in a background of acute and chronic inflammation predominantly composed of neutrophils, lymphocytes, plasma cells and eosinophils (Fig. 1c, e). Calcium and iron accumulates on digested bacterial glycolipids taking on a distinct targetoid or lamellated appearance. These basophilic inclusions are known as Michaelis-Gutmann bodies measuring approximately 4–10 μm in size and are pathognomonic for malakoplakia. They are readily identified on hematoxylin and eosin, von-Kossa, periodic acid Schiff or iron stains (Fig. 2a, b) [1, 6, 7].

Immunosuppression can be caused by medications (i.e. steroids, immunomodulators, chemotherapy, etc.) or disease states (i.e. acquired immune deficiency syndrome, infection, autoimmune conditions, malignancy, post organ transplantation, diabetes mellitus, etc.). Eight patients with malakoplakia were on immunosuppressive medications (8/23, 35%). Common immunosuppressed disease states included cancer (*n* = 9), autoimmune diseases including inflammatory bowel disease (*n* = 5), diabetes (n = 5), status post organ transplantation (*n* = 4), infection or sepsis (*n* = 3), and HIV/AIDS (*n* = 1). Some patients had multiple co-morbidities (i.e. diabetes and organ transplant). Overall, 21 patients with malakoplakia were in an immunosuppressive disease state (21/23, 91%). Of the 9 malakoplakia cases occurring in patients with a malignancy, 3 cancers arose in the colon. The other cases include 2 prostate cancers, 1 bladder cancer, 1 diffuse large B-cell lymphoma, 1 non-Hodgkin’s lymphoma, and 1 invasive thymoma. Two of three colon cancers and the diffuse large B cell lymphoma had malakoplakia within or adjacent to the tumor site. All other cases with an associated malignancy were negative for malakoplakia at or next to the tumor site.

## Discussion

To our knowledge, this is the largest case series of malakoplakia in the gastrointestinal tract. Our results show that it involves the colon with the sigmoid colon and rectum being most frequently involved sites, followed by the descending and transverse colon. The majority of patients are either on an immunosuppressive medication (i.e. steroids, chemotherapy, immunomodulatory agents) or afflicted by a disease (cancer requiring chemotherapy, status post organ transplant, or inflammatory bowel disease) that compromises their immune status. If malakoplakia is found in gastrointestinal mucosal biopsies or resections, the possibility of an immunocompromised status should be communicated to the clinician if immune status is unknown. Recognition of malakoplakia by the pathologist is critical in establishing appropriate patient care because antibiotic therapy (i.e. trimethoprim-sulfamethoxazole, rifampicin and quinolone) is effective and curative. These medications kill the bacteria within the macrophages. Bethanechol chloride is a cholinergic agonist that increases cyclic guanosine levels, increasing the bactericidal activity of impaired lysosomes [1, 2].

The main differential diagnosis on endoscopy is malignancy or premalignant neoplasm (e.g. adenoma) because malakoplakia can present as a polyp, mass, ulceration or involve lymph nodes. It can rapidly grow as single or multiple nodules and may lead to premature or unnecessary surgical intervention. Gastrointestinal tract involvement has also resembled inflammatory bowel disease or tuberculosis on colonoscopy. The microscopic features raise a broad differential. There are numerous conditions with a histiocytic proliferation: xanthoma, Crohn’s disease, Whipple’s disease, granular cell tumor, sarcoidosis, tuberculosis, granulomas from medication effect or fungal organisms, *Mycobacterium avium* infection, melanosis coli, Langerhans cell histiocytosis, Chediak-Higashi syndrome and metabolic storage diseases. The presence of Michaelis-Gutman bodies, particularly if confirmed by calcium stain is highly specific for malakoplakia. Missing a treatable infection could have particularly negative consequences for the patient. Therefore, staining for acid fast bacilli (to exclude *Mycobacterium avium* infection and tuberculosis) and periodic acid Schiff with diastase (to exclude Whipple’s disease) is a reasonable step to pursue before diagnosing malakoplakia. Crohn’s disease, sarcoidosis, medication granulomas, and Chediak-Higashi syndrome all tend to have more epithelioid granulomas and or giant cells. Malakoplakia is more of a sheet-like pattern of histiocytes. Should one remain concerned about non-histiocytic neoplasia, a negative S100 immunostain essentially excludes granular cell tumor, whereas CD1a, S100, and langerin can be employed to exclude Langerhans cell histiocytosis.

Since immunosuppressed patients are predisposed to malakoplakia, pathologists should be aware that more than one disease process may be present. Indeed, this series includes cases with associated cytomegalovirus and amoeba infections (Fig. 2c–e). Malakoplakia has also been described in association with numerous conditions such as tubular adenomas, adenocarcinomas, diverticulosis, ulcerative colitis, neurofibromas, cirrhosis, tuberculosis, stomach ulcers and lymphoid hyperplasia. Intestinal metaplasia of the esophagus (Barrett’s esophagus) is a response to prolonged stomach acid reflux and has also been associated with malakoplakia [1, 23]. Despite the differential considerations discussed above, ultimately, this condition is usually not too challenging to diagnose, if it is considered. Here we have highlighted the clinical associations, affected sites, endoscopic appearance, histologic appearance, and potential complications of malakoplakia in the gastrointestinal tract.

## Data Availability

N/A
